# Organic Farming Practices and Shade Trees Reduce Pest Infestations in Robusta Coffee Systems in Amazonia

**DOI:** 10.3390/life11050413

**Published:** 2021-04-30

**Authors:** Kevin Piato, Cristian Subía, Jimmy Pico, Darío Calderón, Lindsey Norgrove, François Lefort

**Affiliations:** 1 Plants and Pathogens Group, Research Institute Land Nature and Environment, Geneva School of Engineering, Architecture and Landscape (HEPIA), HES-SO University of Applied Sciences and Arts Western Switzerland, 1254 Jussy, Switzerland; 2 School of Agricultural, Forest and Food Sciences (HAFL), Bern University of Applied Sciences (BFH), 3052 Zollikofen, Switzerland; lindsey.norgrove@bfh.ch; 3 National Institute of Agronomical Research (INIAP)-Central Experimental Station of Amazonia, km 3 Vía Sacha-San Carlos, Cantoón, La Joya de los Sachas 220350, Ecuador; cristian.subia@iniap.gob.ec (C.S.); jimy.pico@iniap.gob.ec (J.P.); dario.calderon@iniap.gob.ec (D.C.)

**Keywords:** *Coffea* *canephora*, biocontrol, agroforestry

## Abstract

Coffee agroforestry systems could reconcile agricultural and environmental objectives. While pests and diseases can reduce yield, their interactions with shade and nutrition have been rarely researched, and are particularly lacking in perennial systems. We hypothesized that intermediate shade levels could reduce coffee pests while excess shade could favor fungal diseases. We hypothesized that organic rather than mineral fertilization would better synchronize with nutrient uptake and higher nutrient inputs would be associated with reduced pest and disease damage due to higher plant vigor, yet effects would be less obvious in shaded plots as coffee growth would be light-limited. Using three-year-old trees of *Coffea* *canephora* *var.* Robusta (robusta coffee) in the Ecuadorian Amazon, we compared a full-sun system with four shading methods creating different shade levels: (1) *Myroxylon* *balsamum*; (2) *Inga* *edulis*; (3) *Erythrina* spp.; or, (4) *Erythrina* spp. plus *Myroxylon* *balsamum*. Conventional farming at either (1) moderate or (2) intensified input and organic farming at (3) low or (4) intensified input were compared in a split-plot design with shade as the main plot factor and farming practice as the sub-plot factor. The infestation of the following pests and disease incidences were evaluated monthly during the dry season: brown twig beetle (*Xylosandrus* *morigerus*), coffee leaf miner (*Leucoptera* *coffeella*), coffee berry borer (*Hypothenemus* *hampei*), anthracnose disease (*Colletotrichum* spp.), thread blight (*Pellicularia* *koleroga*), and cercospora leaf spot (*Cercospora* *coffeicola*). Coffee berry borer and brown twig beetle infestation were both reduced by 7% in intensified organic treatments compared to intensified conventional treatments. Colonization of coffee berry borer holes in coffee berries by the entomopathogenic fungus *Beauveria bassiana* was also assessed. Brown twig beetle infestation was significantly higher under full sun than under *Inga edulis,* yet no other shade effects were detected. We demonstrate for the first time how intensified input use might promote pest populations and thus ultimately lead to robusta coffee yield losses.

## 1. Introduction

Current global coffee productivity is threatened by both climate change [1,2,3] and a high price volatility, affecting approximately 17 million coffee farmers, mainly smallholders, worldwide [4,5,6]. Climate change might favor the outbreak of pests and diseases [7], making coffee growers more dependent on external inputs. The trend towards full-sun coffee might also increase reliance on external inputs, although both *Coffea canephora* Pierre ex. A. Froehner and *C*. *arabica* L. can grow under shade [8]. Greater use of fungicides and insecticides might also increase secondary pest problems due to their impacts on beneficial natural enemies, whether insect predators or entomopathogens [9]. Shade-grown coffee crops might be more sustainable [10,11] and might better reconcile agricultural, social, and environmental goals [12].

The total daily assimilation rate of a coffee tree is greater in the shade than in the sun [13,14]. Shade tree inclusion also modifies the microclimate in coffee fields [15,16]. Barradas and Fanjul [17] found that the average maximum temperature in a coffee plantation in Mexico was reduced by more than 5 °C, compared to unshaded coffee, consequently reducing the vapor pressure deficit. Shade tree inclusion can modify air movement and wind in the understory [18]. A recent meta-analysis on the impact of shade trees on robusta coffee showed that shade trees may enhance yield, growth, and biodiversity, yet few studies tested the effects on pests and diseases to allow a general judgment to be made [19]. Arabica and robusta coffee trees do not have the same ecology [20] and recommended fertilizer rates vary [21], which might alter the shade response [10,18,22,23,24], and the type of shade trees used [25]. Furthermore, *C*. *arabica* and *C*. *canephora* may not have the same sensitivity to pests and diseases [26]. Disease incidence can also be modified by soil fertility and by fertilizer inputs. For example, Silva et al. [27] showed that soil P and K correlated positively with coffee leaf rust incidence whereas Ca correlated negatively. Avelino et al. [28] considered that fertilization would modify berry load and leaf area of coffee, and this would in turn modify the coffee risk infection cycle. Staver et al. [9] summarized effects of shade in arabica plantations on various pest and diseases species stating that cercospora had a strongly negative response to shade whereas other fungal pathogens were not affected or had erratic responses. There are few studies available on other coffee diseases. Research is needed to determine if shade provides any pest and disease control and to assess how it interacts with crop management to develop best-bet robusta coffee systems [29].

Ecuador produces both arabica and robusta coffee and is in the top 20 world producers of coffee. However, production has been decreasing from 90,000 metric tons per year in 1991 to 37,000 metric tons in 2018 [30]. In the Ecuadorian Amazon Region (EAR), more than 60% of farmers cultivate coffee, although production is constrained by unsuitable soils, pests and disease losses, and the lack of education and training for farmers [31]. According to Nieto and Caicedo [31], pests and diseases reduce coffee yield by 30% to 40% in the EAR.

In our study, we used three tree species as shade providers: *Myroxylon balsamum* L. (MB), *Erythrina* spp. (ES), and *Inga edulis* Mart. (IE) either alone or in combination, as well as the herbaceous *Musa* spp. AAB (plantain) in all coffee plots. IE produces edible fruit and wood [32]. Tree polycultures might have fewer pest outbreaks and retain more natural enemies [33] but cannot eliminate pest and disease outbreaks [16], as these occur even in highly diverse natural vegetation [34]. Plantain grows fast and provides additional income before the coffee yield is realized. The deciduous fast-growing tree MB of the Fabaceae family is common in tropical forests between 200 m a.s.l. and 690 m a.s.l. [35] but not in the EAR [36]. Nevertheless, its presence could provide an additional amount of notable income because its resin is widely used by the cosmetics and pharmaceutical industry [37]. ES (Fabaceae), an evergreen legume pantropical tree, is widely used throughout the world as a shelter tree, especially in cocoa agroforestry systems [38]. Here we chose to combine MB and ES, an evergreen with a deciduous tree to have constant shade throughout the year, which might improve coffee quality [39].

**Figure 1:**
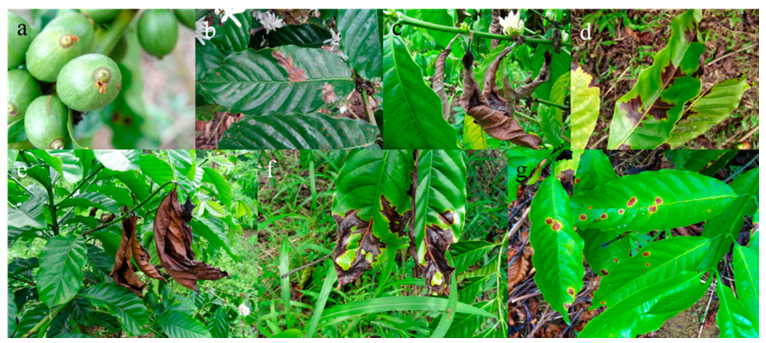
Pests and fungi affecting *Coffea canephora var.* Robusta plants in the Ecuadorian Amazonia (© Kevin Piato): (**a**) *Hypothenemus hampei*, (**b**) *Leucoptera coffeella*, (**c**) *Pellicularia koleroga*, (**d**) *Phoma* spp., (**e**) *Xylosandrus morigerus*, (**f**) *Colletotrichum* spp., and (**g**) *Cercospora coffeicola.*

We aimed to assess pests and diseases under different agroforestry systems and intensive versus organic farming practices at different input levels. We hypothesized that intermediate shade levels, where coffee growth is optimized, could reduce coffee pests while too much shade could favor fungal diseases. We hypothesized that higher nutrient inputs would be associated with reduced pest and disease development due to higher plant vigor and that organic rather than mineral fertilization would lead to further benefits due to the slower nutrient release rates. We hypothesized that any positive effect of nutrients would be less obvious in plots with higher shade levels given that plant growth would be light-limited. Shade level was not expected to be high because all trees were not fully developed. We also estimated shade level in each treatment in order to discriminate between the shading effect of a tree and its specific species interaction with pests and diseases (Figure 1).

## 2. Materials and Methods

### 2.1. Site Description

This study was conducted from July–September 2018 in La Joya de los Sachas in the province of Orellana in Ecuador (latitude 00°21′31.2″ S, longitude 76°52′40.1″ W) at the INIAP’s Amazonian Central Station (EECA). The climate is classified as Af (tropical rainforest climate) according to the Köppen–Geiger climate classification [40]. Based on 37 years of data, the average annual precipitation is between 3000 and 3500 mm in the region of La Joya de los Sachas [41], and the average annual temperature is between 26 °C and 27 °C [42] with an elevation of 250 m above sea level (a.s.l.). Meteorological data during the study is shown in Table 1. In 2018, the dry season began in August 2018, with lower relative humidity (RH), rainfall, and higher temperature. Soil is classified as an Andic Dystrudepts with 43% of clay, 27% of silt, and 30% of sand [43]. Vegetation is humid, moist forest [44]. The year 2018 was representative in terms of rainfall and temperature when compared to the previous 8 years. In 2018, a mean max temperature of 30.66 °C, min temperature of 22.56 °C, and total annual rainfall of 2955 mm were recorded in La Joya de los Sachas [45] which are close, respectively, to the mean max temperature of 30.58 °C, min temperature of 20.33 °C, and rainfall of 3050 mm per year [46] from 2010 to 2017 (Figures S1–S3).

### 2.2. Experimental Design

The experiment had a split-plot design (n = 3) with agroforestry treatment as the main-plot factor and farming practice (fertilization and weeding) treatment as the sub-plot factor. The main plot size was 120 m × 36 m, divided into 4 sub-plots, each 30 m × 36 m. The previous land use was a 9-year-old palm oil plantation and was cleared in 2015. For the shade assessments, we did not consider differences between the sub-plot factors so the data were analyzed as a randomized complete block design (RCBD, n = 4).

On 20th November 2015, *Coffea canephora var.* Robusta plants of the NP-3013 and NP-2024 clones were planted in all plots at 1333 plants per hectare (3 m × 2.5 m spacing) with 100 g of fertilizer (10:30:10) applied by hand on the ground near the stem. These cultivars are not susceptible to the coffee leaf rust pathogen *Hemilia vastatrix*. Different shade treatments were then added to the coffee plots: *Myroxylon balsamum* and *Musa spp.* (TIM), where MB was at 83 stems per hectare (10 m × 12 m spacing); *Erythrina* spp. and *Musa* spp. (ERY), where ES was at 333 stems per hectare (5 m × 6 m spacing); *Inga edulis* and *Musa* spp. (GUA), where IE was at 83 stems per hectare (10 m × 12 m spacing); and *Myroxylon balsamum*, *Erythrina* spp., and *Musa* spp. (TaE), with both MB and ES trees at a total density of 83 stems per hectare and planted alternately. The fifth treatment was a full sun system without trees (SUN). All treatments, except SUN, also contained *Musa* spp. AAB plants as temporary shade at 333 plants per hectare (5 m × 6 m spacing). IE trees were replanted on 24.07.2017 due to poor growth, so they were one year old when the field assessments were performed.

Four farming practices, varying in their fertilizer amounts, types, and weeding were compared as sub-plot treatments: intensive conventional (IC); moderate conventional (MC); intensive organic (IO); and low organic (LO). In IC and MC farming practices, the fertilizer amounts applied in week 20 of 2018 were: 34 kg/ha of KNO_3_, 41 kg/ha of KH_2_PO_4_, 51 kg/ha of YaraMila Actyva 20-7-10-2MgO-10SO_3_, 123 kg/ha of YaraMila Hydran 19-4-19-3MgO, and 41 kg/ha of NH_4_NO_3_. This corresponded to 225 g of chemical fertilizer per coffee plant. In IO and LO farming practices, 1334 kg/ha of Eco Abonaza India 3.5-2-3.5 (a mix of semi-composted chicken manure and sawdust with a content of 70–73% of organic matter) were applied in week 9 of the year 2018. This corresponded to 1000 g of organic fertilizer per coffee plant. In all farming practices, fertilizer was applied to the side of the coffee plant by hand.

Farming practices also differed in the type and frequency of weeding. Weeds were controlled by means of herbicides, with 4 applications per year in IC plots against 2.5 in MC plots. Furthermore, mechanical weeding with brush-cutters was done, with respectively 4, 4, 5, and 4 interventions in IC, MC, IO, and LO plots (Table S1). All mechanical weeding was done with a string strimmer and residue was left in place.

### 2.3. Pest and Disease Sampling

Incidences of the following were evaluated monthly on coffee: the brown twig beetle, *Xylosandrus morigerus* (BTB); the coffee leaf miner, *Leucoptera coffeella* (CLM); the coffee berry borer, *Hypothenemus hampei* (CBB); anthracnose disease, *Colletotrichum* spp. (AD); thread blight, *Pellicularia koleroga* (TB); cercospora leaf spot, *Cercospora coffeicola* (CLS); and phoma leaf spot, *Phoma* spp. (PLS). Furthermore, AD severity was assessed on fruits damaged by CBB. Finally, the presence of the entomopathogen *Beauveria bassiana* (BB) was detected by the presence of white mycelium covering the holes made by CBB. All pest and disease assessments were made on the 36 central square coffee plants only (6 × 6 plants) per subplot. Nine plants were randomly selected from the central area, per plot, across the various shade zones. Thus, 540 plants were assessed in the 60 plots. As the focus of the study was on coffee pests and diseases, we did not make such measurements on the shade trees.

The following symptoms were used to discriminate between diseases and pests tested: necrotic brown-black lesions in the margin of the leaf with a dry appearance for AD; malformation of young leaves with brown spot with a shiny appearance for PLS; circular small brown spots with a center tan to light brown for CLS; overleaf blackening and rotting leading to leaf detachment which remains attached through fungal strand for TB; presence of a hole near the apex of the fruit, sometimes with sawdust around it for CBB; presence of leaf lesions between the epidermis showing two visible layers for CLM; presence of twig necrosis with one or several holes for BTB [8,47].

We measured all variables on 12–13 July 2018, 13–14 August 2018, and 3 September 2018 (except irradiation measurements that were made once), and AD severity was assessed on 12–13 July 2018 only. For each robusta coffee plant selected, 3 branches were randomly selected at <50 cm, 50–100 cm, and >100 cm height. The short internode was identified in each branch to distinguish between the current year’s leaves and those of the previous year. All healthy leaves from the short internode to the branch tip of the branch were counted. Leaves infected by AD, PLS, CLS, TB, and infested by CLM were also counted by section. We sampled two leaves per branch (those after the short internode) to determine AD severity, for a total of 6 leaves per plant (according to [48]). This method was used as older leaves are more likely to be infested and infected, senescing more easily than younger ones. All branches on one coffee stem were counted, as well as all branches with BTB damage. Moreover, all the fruit on two other branches randomly selected at <50 cm and 50–100 cm height but displaying enough fruit were counted: one from the low section and one from the middle section. Then all the fruit showing a CBB hole and BB presence were counted.

### 2.4. Shade Estimates and Solar Radiation Measurements

Total irradiation measurements above coffee plants were made with an Apogee MP-200 silicon-cell pyranometer with a separate sensor and a levelling plate. Shade was defined as the quantity of total solar radiation in W/m^2^ that cannot reach the coffee plants since it is absorbed or reflected by the overstory trees. Shade measurements were made only in one block due to time limitations. Solar radiation was measured in the central part of the plot, comprising 6 × 6 coffee plants, to avoid edge effects. For each of the shade types, except for the SUN, zones between shade trees were defined (Table S2) to take account of heterogeneous shade distribution within the plot. Solar radiation was measured with a pyranometer on 1 randomly selected coffee plant per zone (Table S2), except for the SUN shade type, in which 2 coffee plants were randomly selected. As 18 zones were defined, 76 coffee plants were assessed in total to work out the shade percentage for each zone (4 × 19 coffee plants).

For each selected coffee plant, 5 measurements of solar radiation were made with the pyranometer: at the apex, the east, west, north, and south of the plant. A reference was also taken in SUN. To avoid interference of self-shading coffee bushes [49], measurements were done at the tip of the branches and at 2 m height. Pyranometer measurements were taken in full sunlight. Measurements were done at 0900h–1030h, 1130h–1300h, and 1400h–1530h.

### 2.5. Determination of Colletotrichum spp. Severity by Image Processing

Leaves collected (3240) were scanned with the multi-function printers EPSON L555 Series and Hewlett-Packard HP Color Laser Jet CM1312 MFP at a resolution of 200 dpi. Images were processed with “ImageJ 1.52a” [50] to determine total leaf area and the total leaf necrotic area. To determine the leaf area damaged by AD only, the following filter of the function “Adjust color balance” was used: Lab-white, L* = 7–95/a*= 124–243/b* = 0–255. Only necrotic areas larger than 500 pixels were considered.

### 2.6. Data Processing

#### 2.6.1. Data Quality Control

Only the total numbers of branches, fruits, and leaves between the 10th and 90th percentile were considered, but not outliers. For mean values of infestation, incidence, and presence rates, only values between 5th and 95th percentiles were considered, and outliers were deleted. These last two steps were also necessary to exclude errors due to the misidentification of the short internode and the bias of selectors taking branches with few fruits to gain time. Data quality control to remove outliers was performed for all variables except AD severity. For AD severity rate, rates between the 1st and 99th percentiles were taken into consideration, and all other values deleted to exclude leaves damaged after the harvest or picked below the short internode.

#### 2.6.2. Calculations

Formula used to calculate all the response variables (except the shading percentage) are outlined in Table 2.

#### 2.6.3. Determination of Shade Percentage

Shade % was calculated with the following formula for each of the 76 coffee plants assessed: shade % = (1 − (average W under shade/W in shade-free)) × (100). This was used to calculate the mean shade percentage per zone. The weighted average shade % of the plot was estimated as: [((average shade percentage of SUN) × (net area percentage of SUN)) + ((average shade percentage of MB_1.95_) × (net area percentage of MB_1.95_)) + … + ((average shade percentage of MB_7.60_ES_4.67_) × (net area percentage of MB_7.60_ES_4.67_))] × (100) for each period of time referred to in Section 2.4.

The daily average shade % of the plot was estimated by taking the mean of the weighted average shade % of each time slot.

### 2.7. Statistical Analysis

Data were analyzed in Minitab^®^ 18 (State College, PA, USA) [51], using mixed linear models for a split-plot design with 3 repetitions, treatments as fixed effects, blocks as random effects, and including the interaction term. Tukey’s test was used for multiple comparisons with a significance level of α = 0.05. Residual normality checks of homogeneity of variance were done prior to analysis and log-transformed, if required. For the shade % data, if the homogeneity variance criterion was not fulfilled, the Friedman non-parametric test was applied to illustrate a difference between the treatments. To compare the average shade % of the 3 periods in a day, the same RCBD was applied, the 3 factors being the shading method, the period in a day, and the block.

## 3. Results

**Figure 2:**
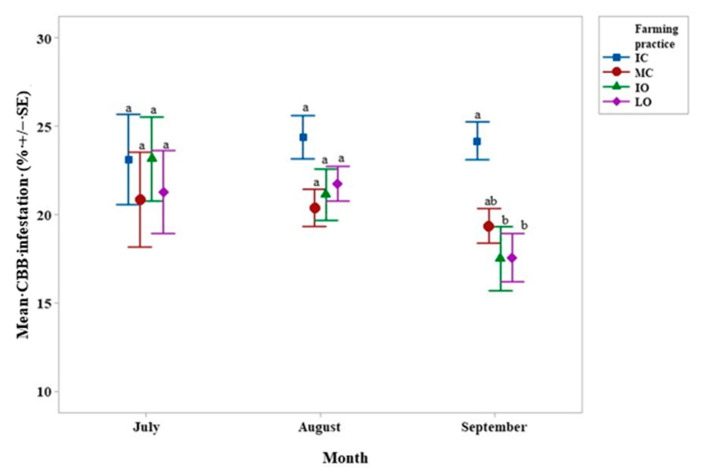
Interval plot of the mean *Hypothenemus hampei* (CBB) infestation (%) according to the month and the farming practice. Values labeled with the same letter are not significantly different at *p* = 0.05. Lines denote 1 s.e. mean. IC = intensive conventional, MC = moderate conventional, IO = intensive organic, LO = low organic.

No significant interactions were detected between shading and farming practices. IO and LO farming practices had a lower mean CBB infestation than the IC farming practice in September only with no significant differences on other dates (Figure 2).

**Figure 3:**
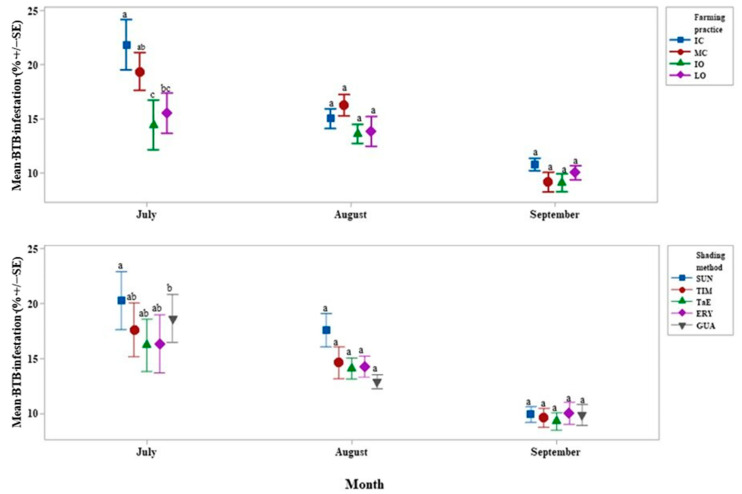
Interval plot of the mean *Xylosandrus morigerus* (BTB) infestation (%) according to the month and the farming practice (above) or the shading method (below). Values labeled with the same letter are not significantly different at *p* = 0.05. Lines denote 1 s.e. mean. IC = intensive conventional, MC = moderate conventional, IO = intensive organic, LO = low organic, SUN = full sun, TIM = *Myroxylon balsamum* and *Musa* spp., TaE = *Myroxylon balsamum*, *Erythrina* spp. and *Musa* spp., ERY = *Erythrina* spp. and *Musa* spp., GUA = *Inga edulis* and *Musa* spp.

The IC treatment had a BTB infestation 7% higher (*p* < 0.01) than the IO farming practice, and 6% higher (*p* < 0.01) than the LO farming practice in July (Figure 3). In July only, the MC treatment had a mean BTB infestation 5% higher (*p* < 0.05) than the IO farming practice in July only. All other differences were insignificant. The SUN treatment had a BTB infestation 5% higher (*p* < 0.05) than the GUA shade treatment in July only. All other differences were insignificant. Over all treatments, mean BTB infestation decreased over time.

**Figure 4:**
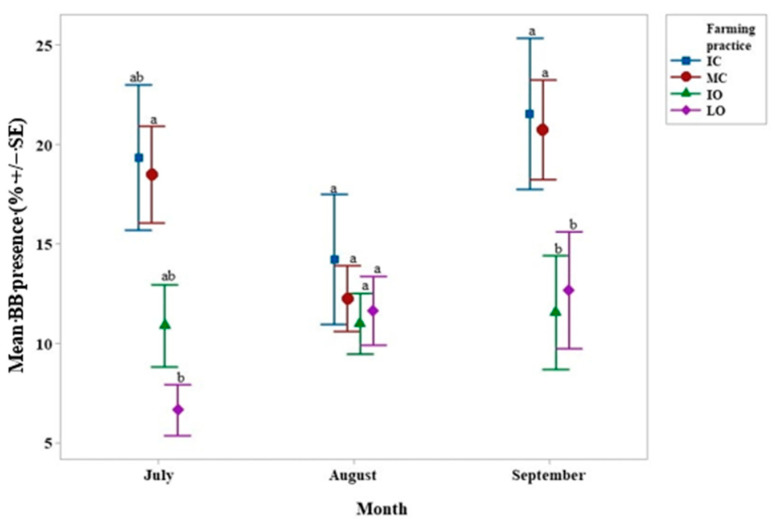
Interval plot of the mean *Beauveria bassiana* (BB) presence (%) according to the month and the farming practice. Values labeled with the same letter are not significantly different at *p* = 0.05. Lines denote 1 s.e. mean. IC = intensive conventional, MC = moderate conventional, IO = intensive organic, LO = low organic.

LO and IO farming practices presented a markedly lower mean BB presence than the IC and MC farming practices for both July and September (Figure 4). According to Tukey’s post-hoc tests, MC farming practice had a mean BB presence 3 times higher (*p* < 0.01) than the LO farming practice in July. In September, the IC farming practice had a mean BB presence 10% higher (*p* < 0.01) than the IO farming practice, and 9% higher (*p* < 0.05) than the LO farming practice. In addition, the MC farming practice had a mean BB presence 9% higher (*p* < 0.01) than the IO farming practice and 8% higher (*p* < 0.05) than the LO farming practice. In August, there were no significant differences between the treatments. 

**Figure 5:**
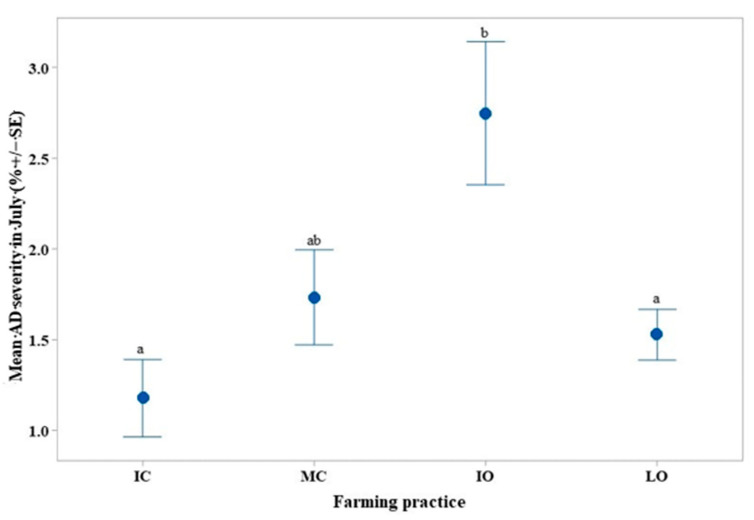
Interval plot of the mean *Colletotrichum* spp. (AD) severity (%) according to the farming practice in July 2018. Values labeled with the same letter are not significantly different at *p* = 0.05. Lines denote 1 s.e. mean. IC = intensive conventional, MC = moderate conventional, IO = intensive organic, LO = low organic.

In July, the IO farming practice had a mean AD severity 1.57% higher (*p* < 0.05) than the IC farming practice, in addition to a mean AD severity 1.22% higher (*p* < 0.05) than the LO farming practice (Figure 5), whereas the IO practice was not significantly different from the MC practice. 

**Figure 6:**
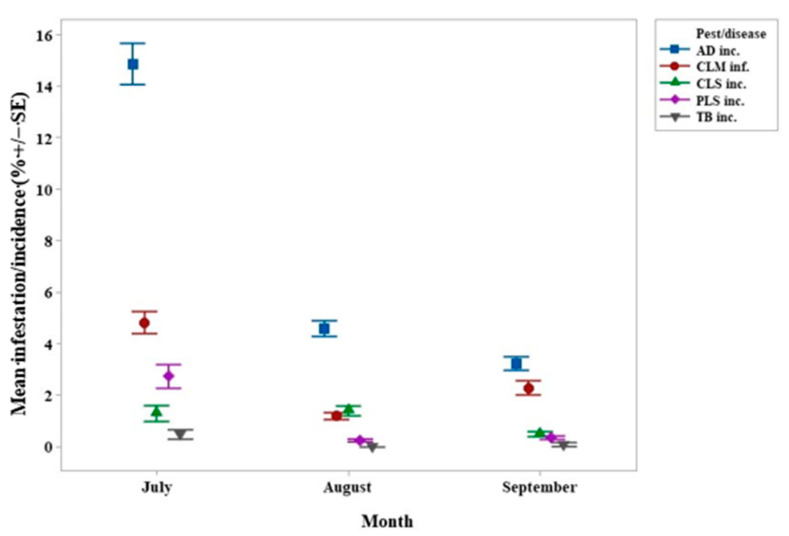
Interval plot of the overall mean *Colletotrichum* spp. incidence (AD inc.), *Leucoptera coffeella* infestation (CLM inf.), *Cercospora coffeicola* incidence (CLS inc.), *Phoma* spp. incidence (PLS inc.), and *Pellicularia koleroga* incidence (TB inc.) in July, August, and September 2018. Lines denote 1 s.e. mean.

The interval plot shown in Figure 6 gathers the overall means for each month of all response variables without any significant results. The mean AD incidence, the mean CLM infestation, and the mean CLS incidence were the lowest in September. Nevertheless, the mean AD incidence increased from July to August and decreased markedly from August to September. 

Mean daily shade percentages of the plots applying the SUN, TaE, TIM, GUA, and ERY shading methods were 0.6%, 9.2%, 15.1%, 26.4%, and 29.8%, respectively (Figure S4). The temporary *Musa* spp. shade is included in the shade percentages achieved. In fact, along with the TIM shading method, the bananas were the main contributors to shade at this early stage in the plot. The same tendency appears in the TaE shading method, where the density of MB trees is 41 plants/ha, whereas it is 83 plants/ha in the TIM shading method. In the TaE shading method, MB trees could not provide so much shade due to the low density, low height, and crown diameter. As to the ES trees within TaE plots, the shade they provided accounted for about 3.7%, and the ERY shading method counted about 333 plants/ha for a 29.8% shade percentage, also meaning that the shade provided by banana trees can be estimated as between 5.5% and 15.1%.

## 4. Discussion

### 4.1. Hypothenemus Hampei Infestation–Beauveria Bassiana Presence

The most compelling effect of the farming practices was on CBB infestation and BB presence in September, which were positively correlated. CBB infestation was much greater in the IC treatment than in the others and BB was much greater in the two conventional treatments (Figure 2 and Figure 4). Similar results have been found in an arabica coffee plantation in Nicaragua where organic and integrated farm managements allowed the reduction of the CBB adult population [52]. Herbicide use was highest in the IC treatment (K. Piato, pers. obs.). As certain weeds such as *Lantana camara*, a common weed in coffee fields [53], can repel CBB, this might explain the lower CBB infestation in the less intensive treatments. Weed removal can reduce ant populations, which are natural enemies of CBB, thus inadvertently increasing infestation [54]. Seven or more ant genera can prey on infested berries and can reduce CBB infestation by up to 27% [55,56,57]. It is crucial to retain complex vegetation within agroecosystems, providing habitats for several ant genera. For instance, the CBB predator, *Pheidole synanthropica*, nests on the ground, whereas the CBB predator *Azteca* sp. needs trees to nest and forage [58].

CBB and BB infestation were also positively correlated (*p* < 0.05 in August and *p* < 0.01 in September). Similar correlations have been found in Mexico [59]. This could explain why significantly less BB was found in CBB-infested fruits in organic treatments (IO or LO) than in conventional ones (IC and MC). Rain splash dispersal may be more pronounced in conventional plots, due to less weed cover, and this could explain why BB was more widespread, as shown elsewhere [60,61]. Pohlan et al. [62] showed that cover crops can reduce CBB populations, since they provide habitats for natural enemies. This hypothesis could explain why there were more CBB, and therefore associated BB, under conventional treatments, whereas the contrary could have been expected [63]. Moreover, cultivation generally does not seem to affect the level of the presence of BB in soils, whether natural or cultivated [64].

Unlike in previous studies [23,29], the CBB infestation was not impacted by shade most probably because the shade was not uniformly distributed within the plots. There were zones within the plots subject to direct sunlight despite the presence of shelter trees. Small sun-exposed patches could favor CBB activity owing to the higher temperature there [65].

### 4.2. Xylosandrus Morigerus Infestation

The BTB and other *Xylosandrus* spp. preferably attack weak coffee plants lacking fertilization, since fertilization reduces the development of ambrosia fungus, associated with BTB feeding [66,67]. Herbicide use in IC and MC practices, through reducing weed biomass, may have reduced natural enemies, especially BB and ants of *Crematogaster, Leptothorax, Pheidole, Pseudomyrmex,* and *Solenopsis* genera [67]. In particularly, a field cage trial performed with robusta coffee twigs showed that *Pheidole megacephala*, commonly found in both the tropics and subtropics, preys on all life stages of *X*. *compactus* [68]. Many coffee weeds are BTB host plants, notably *Verbena littoralis,* which is found in Ecuador [69]. Removing weeds might have increased BTB infestation, as it is no longer diluted among alternative hosts.

A reduction in *X*. *compactus* infestation with shade was demonstrated in Uganda [25]. However, in our study, the ERY treatment had higher shade yet BTB infestation was not different from that in the shade-free control. Alternatively, as IE is a host for BTB [69], it also might attract BTB, reducing its impact on robusta coffee plants. Further research is needed to firmly corroborate this hypothesis.

### 4.3. Colletotrichum spp. (AD) Incidence and Severity

AD spreads through water-borne conidia, maximum growth occurring at pH 6.5–7.0 and 25 °C–30 °C; conidia germinate at 95%–100% relative humidity (RH) [70,71,72]. In mid-July, AD incidence was high (Figure 6), due to a favorable climate for fungus in June, with RH of 95% at 7 am allowing the conidia to germinate (Table 1). In June, the mean maximum temperature did not exceed 30 °C, another factor contributing to a better growth of AD. AD incidence decreased significantly in August (Figure 6), after the mean maximum temperature had exceeded 30 °C in July and RH remained above 95% (Table 1). The decrease continued in September, after the temperature had exceeded 30 °C in August, when it rained less (150.7 mm) and the RH decreased so these changes may explain the lower AD incidence in September (Table 1). The impact of shade treatment on AD was not significant, whereas AD had the highest severity in the IO treatment with other farming practices being variable.

### 4.4. Shade Percentages

CBB infestation responds to shade levels > 25% [29]. As coffee plants were at the sapling stage, they did not provide self-shading, and the MB trees had been pruned and were slow growing. Therefore, MB trees did not contribute much to the shade % so plantains were the main shade providers. Plantains provided varying amounts of shade depending on height and leaf number. However, given a quadrupled tree density, the ERY shading method had the highest shade % (30%). Nevertheless, the IE shading method with almost the same shade % as the ERY one could be more practical for farmers. Using it would reduce pruning requirements, as the tree density of IE shading method is half as high as for the ERY one. Additionally, according to on-site observations, no shelter trees suffered from pest or disease damage to a degree able to reduce substantially the shade amount.

## 5. Conclusions

In young coffee farms under different shade and farming practices, infestation rates of the coffee berry borer and the brown twig beetle were significantly and up to 7% higher when more intensive and conventional practices were applied, compared with organic practices at the same input level or lower intensity. The presence of the entomopathogen *Beauveria bassiana* was higher in the conventional intensive plots but this was probably related to the higher infestation of the pests. Brown twig beetle infestation was also higher in shade-free conditions; yet on the contrary, shade-free conditions reduced anthracnose severity, albeit only marginally (<2%). This highlights a trade-off between pest and disease damage when comparing shaded and unshaded conditions although the reduction in pest infestation with shading was greater than the increase in fungal diseases. While this study demonstrates pest and disease dynamics at an early stage in the plantation life, it highlights that intensified conventional input use, while potentially leading to high plant productivity, might promote pest populations and thus ultimately lead to greater yield losses.

## Figures and Tables

**Table 1:** Mean maximum, minimum temperatures, total rainfall, and mean relative humidity (RH) per month at 07 h, 13 h, and 19 h at La Joya de los Sachas meteorological station, Ecuador [45].

Month	Mean Max Temp. (°C)	Mean Min Temp. (°C)	Total Rainfall (mm)	Mean RH at 7 am (%)	Mean RH at 1 pm (%)	Mean RH at 7 pm (%)
April 18	30.1	20.8	299.0	95	80	85
May 18	30.2	22.0	358.9	90	80	90
June 18	29.5	21.8	193.5	95	80	85
July 18	30.3	21.4	212.9	90	80	85
August 18	30.7	21.7	150.7	90	75	80
September 18	32.1	22.2	114.6	90	70	80

**Table 2:** Formulae for pest, disease, and growth variables.

Response Variables (%)	Variables	Meaning	Formula
CBB inf. ^1^	*n’*	Number of cherries where CBB was present	(*n’*/*N’*) ×100
	*N’*	Total number of cherries assessed	
AD inc. ^2^	*N*	Number of infected leaves	(*n*/*N*) × 100
PLS inc.			
CLS inc.	*N*	Total number of leaves	
TB inc.			
BTB inf.	*R*	Number of branches where BTB was present	(*r*/*R*) × 100
CLM inf.	*R*	Total number of branches assessed	
AD sev. ^3^	*S*	Total necrotic area of leaves	(*s*/*S*) × 100
	*S*	Total area of leaves	
BB pres. ^4^	*B*	Number of cherries perforated by CBB with BB	(*b*/*B*) × 100
	*B*	Total number of cherries perforated by CBB	

^1^ Infestation; ^2^ Incidence; ^3^ Severity; ^4^ Presence.

## Data Availability

The data presented in this study are available on request from the corresponding author.

## Supplementary Materials

The following materials are available online at https://www.mdpi.com/article/10.3390/life11050413/s1, Figure S1: Rainfall per year (mm) from 2010 to 2017 in La Joya de los Sachas, Orellana, Ecuador. Figure S2: Max. temperature per year (°C) from 2010 to 2017 in La Joya de los Sachas, Orellana, Ecuador. Figure S3: Min temperature per year (°C) from 2010 to 2017 in La Joya de los Sachas, Orellana, Ecuador. Figure S4: Box and whisker plots of the 2018 mean shade (%) in relation to the shading method. Table S1: Weeding schedule in 2017 and 2018 for IC, MC, IO and LO farming practices with herbicide rates (L ha^−1^) in parentheses. Table S2: Defined zones with homogeneous shade, according to distance from coffee plants to shelter trees.
