# Supplementary material for: Organic Farming Practices and Shade Trees Reduce Pest Infestations in Robusta Coffee Systems in Amazonia
Source: Life (Basel). 2021 Apr 30;11(5):413. doi: 10.3390/life11050413 (PMC8147205; doi:10.3390/life11050413)
Supplement: Supplementary file 1 [file life-11-00413-s001.zip › life-1140042-supplementary.pdf]

life

MDPI

Article

Organic Farming Practices and Shade Trees Reduce Pest Infestations in Robusta Coffee Systems in Amazonia

Kevin Piato [1,3,*], Cristian Subía [2], Jimmy Pico [2], Darío Calderón [2], Lindsey Norgrove [3] and François Lefort [1,*]

[1] Plants and Pathogens Group, Research Institute Land Nature and Environment, Geneva School of Engineering, Architecture and Landscape (HEPIA), HES-SO University of Applied Sciences and Arts Western Switzerland, 1254 Jussy, Geneva, Switzerland

[2] National Institute of Agronomical Research (INIAP)-Central Experimental Station of Amazonia, km 3 Vía Sacha-San Carlos, Cantoón, 220350 La Joya de los Sachas, Ecuador; cristian.subia@iniap.gob.ec (C.S.); jimy.pico@iniap.gob.ec (J.P.); dario.calderon@iniap.gob.ec (D.C.)

[3] School of Agricultural, Forest and Food Sciences (HAFL), Bern University of Applied Sciences (BFH), 3052 Zollikofen, Switzerland; lindsey.norgrove@bfh.ch

* Correspondence: kevin.piato@students.bfh.ch (K.P.); francois.lefort@hesge.ch (F.L.)

Abstract: Coffee agroforestry systems could reconcile agricultural and environmental objectives. While pests and diseases can reduce yield, their interactions with shade and nutrition have been rarely researched, and are particularly lacking in perennial systems. We hypothesized that intermediate shade levels could reduce coffee pests while excess shade could favor fungal diseases. We hypothesized that organic rather than mineral fertilization would better synchronize with nutrient uptake and higher nutrient inputs would be associated with reduced pest and disease damage due to higher plant vigor, yet effects would be less obvious in shaded plots as coffee growth would be light-limited. Using three-year-old *Coffea canephora* (robusta coffee) in the Ecuadorian Amazon, we compared a full-sun system with four shading methods creating different shade levels: 1) *Myroxylon balsamum*; 2) *Inga edulis*; 3) *Erythrina* spp.; or, 4) *Erythrina* spp. plus *Myroxylon balsamum*. Conventional farming at either 1) moderate or 2) intensified input and organic farming at 3) low or 4) intensified input were compared in a split-plot design with shade as the main plot factor and farming practice as the sub-plot factor. The infestation of the following pests and disease incidences were evaluated monthly during the dry season: brown twig beetle (*Xylosandrus morigerus*), coffee leaf miner (*Leucoptera coffeella*), coffee berry borer (*Hypothenemus hampei*), anthracnose disease (*Colletotrichum* spp.), thread blight (*Pellicularia koleroga*), and cercospora leaf spot (*Cercospora coffeicola*). Coffee berry borer and brown twig beetle infestation were both reduced by 7% in intensified organic treatments compared to intensified conventional treatments. Colonization of coffee berry borer holes in coffee berries by the entomopathogenic fungus *Beauveria bassiana* was also assessed. Brown twig beetle infestation was significantly higher under full sun than under *Inga edulis*, yet no other shade effects were detected. We demonstrate for the first time how intensified input use might promote pest populations and thus ultimately lead to robusta yield losses.

Keywords: *Coffea canephora*; biocontrol; agroforestry

Citation: Piato, K.; Subía, C.; Pico, J.; Calderón, D.; Norgrove, L.; Lefort, F. Organic Farming Practices and Shade Trees Reduce Pest Infestations in Robusta Coffee Systems in Amazonia. *Life* **2021**, *11*, 413. https://doi.org/10.3390/life11050413

Academic Editor: Balazs Barna and Gustavo Caetano-Anolles

Received: 23 February 2021
Accepted: 19 April 2021
Published: 30 April 2021

Publisher's Note: MDPI stays neutral with regard to jurisdictional claims in published maps and institutional affiliations.

Copyright: © 2021 by the authors. Submitted for possible open access publication under the terms and conditions of the Creative Commons Attribution (CC BY) license (http://creativecommons.org/licenses/by/4.0/).

Supplementary Material

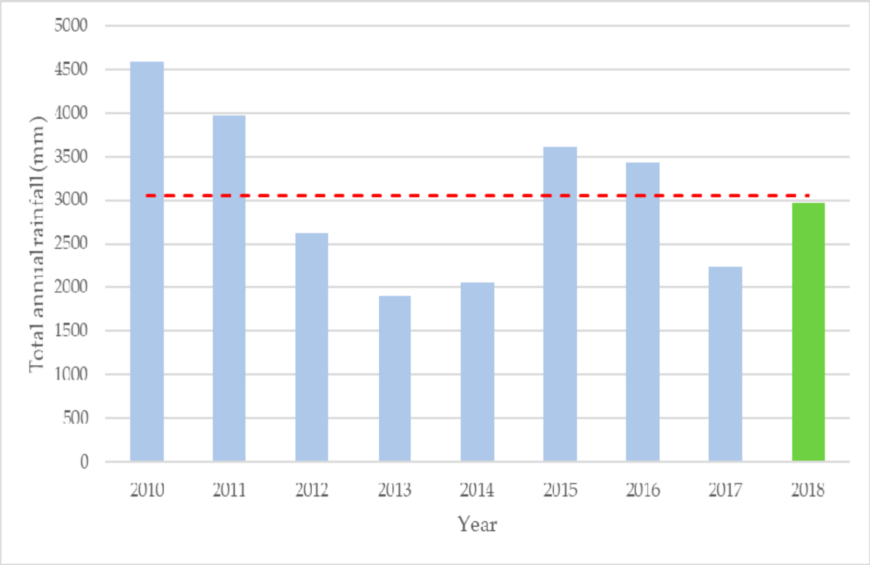

Figure S1. Total annual rainfall (mm) from 2010 to 2018 in La Joya de los Sachas, Orellana, Ecuador. The red dashed line shows the average rainfall per year from 2010 to 2017 [46].

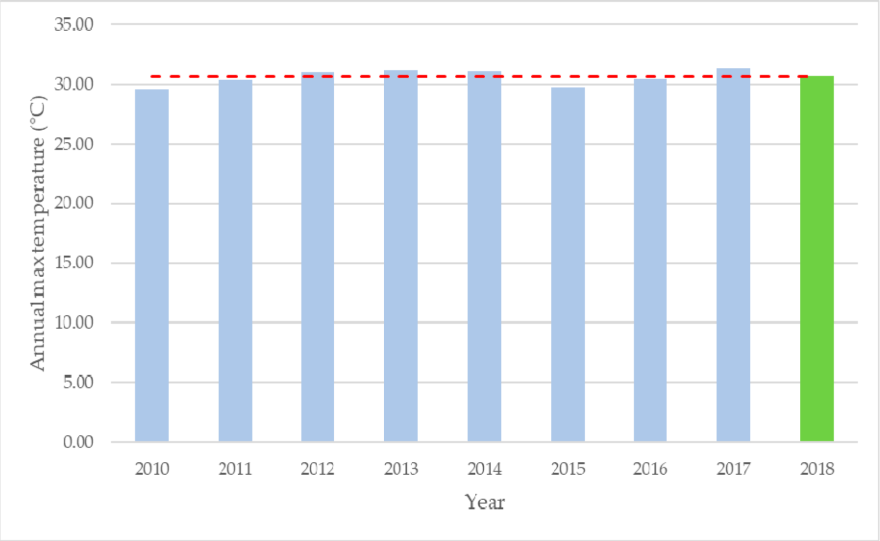

Figure S2. Annual max temperature (°C) from 2010 to 2018 in La Joya de los Sachas, Orellana, Ecuador. The red dashed line shows the average max temperature per year from 2010 to 2017 [46].

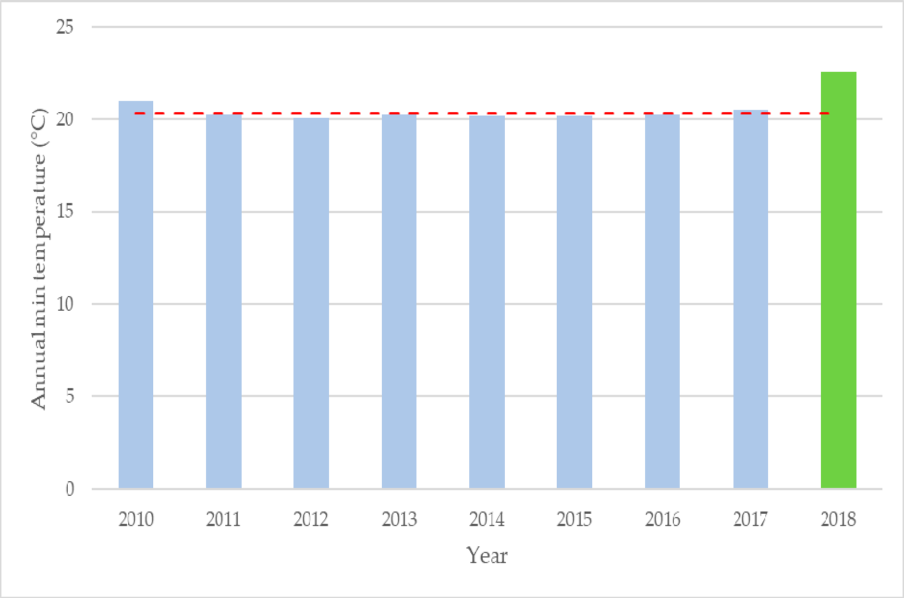

Figure S3. Annual min temperature (°C) from 2010 to 2018 in La Joya de los Sachas, Orellana, Ecuador. The red dashed line shows the average min temperature per year from 2010 to 2017 [46].

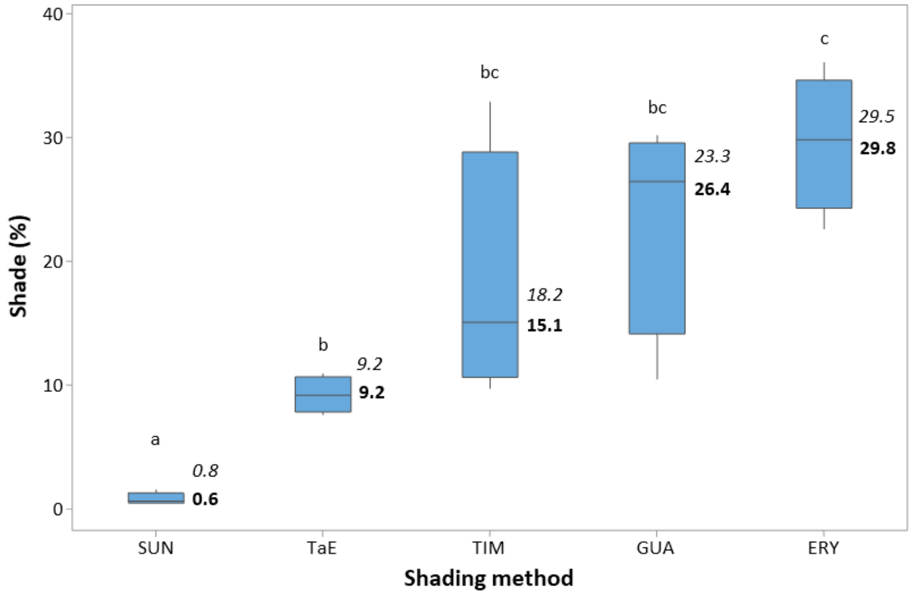

Figure S4. Box and whisker plots of the 2018 mean shade (%) in relation to the shading method. Values labelled with the same letter are not significantly different at p=0.05, SUN= full sun, TIM= *Myroxylon balsamum* and *Musa* spp., TaE= *Myroxylon balsamum*, *Erythrina* spp. and *Musa* spp., ERY= *Erythrina* spp. and *Musa* spp., GUA= *Inga edulis* and *Musa* spp.

Table S1. Weeding schedule in 2017 and 2018 for IC, MC, IO and LO farming practices with herbicide rates (L ha^{-1}) in parentheses.

**Week Number 2017**	**Farming Practice**							
	**IC**		**MC**		**IO**		**LO**	
	**Weeding method**							
	**CHEM**[a]	**MECH**[b]	**CHEM**	**MECH**	**CHEM**	**MECH**	**CHEM**	**MECH**
34	✓		✓					
35	(3.46[c])		(3.46[c])					
41		✓		✓				✓
43	✓ (1.56[c])							
44			✓ (1.73[c])					
45						✓		
48	✓ (1.48[c])							
50						✓		
52				✓				✓
Week Number 2018								
2	✓ (2.3[c])					✓		
3			✓ (1.44[c])					
7			✓ (1.3[c])					
12						✓		
14	✓ (0.92[c])							
15				✓				✓
20	✓ (0.64[d])							
24	✓ (0.64[d])			✓				✓
26						✓		
27		✓	✓ (1.4[c])					
28	✓ (0.64[d])							
34		✓						
41		✓						
Sum	8	4	5	4	0	5	0	4

[a]Chemical weeding; [b]Mechanical weeding; [c]Paraquat; [d]Goal Tender.

Table S2. Defined zones with homogeneous shade, according to distance from coffee plants to shelter trees. # coffee plants sampled per zone ∝ % plot in that zone.

Name of Zone	Shading Method	Net Area %	Distance between Coffee Plants and Shelter Trees (m)	Coffee Plants to Sample
SUN	SUN	100	-	36
$MB_{1.95}$	TIM	11.25	1.95 from *Myroxylon balsamum*	4
$MB_{4.04}$	TIM	22.25	4.04 from *M. balsamum*	8
$MB_{4.67}$	TIM	22.25	4.67 from *M. balsamum*	8
$MB_{5.86}$	TIM	44.25	5.86 from *M. balsamum*	16
$IE_{1.95}$	GUA	11.25	1.95 from *Inga edulis*	4
$IE_{4.04}$	GUA	22.25	4.04 from *I. edulis*	8
$IE_{4.67}$	GUA	22.25	4.67 from *I. edulis*	8
$IE_{5.86}$	GUA	44.25	5.86 from *I. edulis*	16
$ES_{1.95\&4.03P}$	ERY	50	1.95 from *E.* spp.<br>4.03 from *E.* spp. pollarded	18
$ES_{1.95P\&4.03}$	ERY	50	1.95 from *E.* spp. pollarded<br>4.03 from *E.* spp.	18
$MB_{1.95}ES_{8.88}$	TaE	11.1	1.95 from *M. balsamum*<br>8.88 from *E.* spp.	4
$MB_{4.04}ES_{6.43}$	TaE	11.1	4.04 from *M. balsamum*<br>6.43 from *E.* spp.	4
$MB_{4.67}ES_{7.60}$	TaE	11.1	4.67 from *M. balsamum*<br>7.60 from *E.* spp.	4
$MB_{7.70}ES_{5.86}$	TaE	22.25	7.70 from *M. balsamum*<br>5.86 from *E.* spp.	8
$MB_{5.86}ES_{7.70}$	TaE	22.25	5.86 from *M. balsamum*<br>7.70 from *E.* spp.	8
$MB_{6.43}ES_{4.04}$	TaE	11.1	6.43 from *M. balsamum*<br>4.04 from *E.* spp.	4
$MB_{7.60}ES_{4.67}$	TaE	11.1	7.60 from *M. balsamum*<br>4.67 from *E.* spp.	4
